# Characteristics of Metakaolin-Based Geopolymer with Cathode Ray Tube Glass

**DOI:** 10.3390/polym13071149

**Published:** 2021-04-03

**Authors:** Marcin Górski, Natalia Wielgus, Krzysztof Loska, Michał Kozioł, Marcin Landrat, Waldemar Ścierski, Krzysztof Pikoń

**Affiliations:** 1 Department of Structural Engineering, Faculty of Civil Engineering, The Silesian University of Technology, Akademicka 5, 44-100 Gliwice, Poland; natalia.paszek@polsl.pl; 2 Department of Water and Wastewater Engineering, Faculty of Energy and Environmental Engineering, The Silesian University of Technology, Akademicka 2, 44-100 Gliwice, Poland; krzysztof.loska@polsl.pl; 3 Department of Technologies and Installations for Waste Management, Faculty of Energy and Environmental Engineering, The Silesian University of Technology, Konarskiego 18, 44-100 Gliwice, Poland; michal.koziol@polsl.pl (M.K.); marcin.landrat@polsl.pl (M.L.); waldemar.scierski@polsl.pl (W.Ś.); krzysztof.pikon@polsl.pl (K.P.)

**Keywords:** geopolymer, CRT glass, metakaolin, strength, curing conditions, development of temperature inside material, toxic metals leaching

## Abstract

Geopolymers can be treated as an environmentally friendly alternative for concrete and enables utilization of various wastes. This paper focuses on the possibility of application of discarded cathode ray tube (CRT) glass inside a metakaolin-based geopolymer in the form of an aggregate, providing an ecological method of recycling of this hazardous material. The main goal of this paper was to develop an optimal composition of a new geopolymer and to describe its behavior under varying curing conditions. A geopolymer made of different mixtures was subjected to flexural and compressive strength tests. The density, mass loss, temperature changes, and metals leaching were determined as well. The results demonstrated that neither the content of CRT glass nor the curing regime has a significant influence on the mechanical behavior. However, the strength of the geopolymer containing 50% CRT glass by mass increased with time in contrast to a geopolymer with a higher CRT glass content. The development of temperature inside the mixture was dependent on the amount of metakaolin. The concentration of toxic metals in an aqueous extract decreased considerably after the encapsulation of CRT glass inside the geopolymer. The presented results indicate that discarded CRT glass can be considered an aggregate for a metakaolin-based geopolymer. The new material shows high strength and makes the CRT glass safe for the environment.

## 1. Introduction

### 1.1. CRT Glass and Its Recycling Methods

Cathode ray tube glass (CRT glass) was commonly used in the past for the production of television sets and computer’s screens. The safe disposal of this product is a serious problem. CRT glass is classified as a hazardous material due to the high lead content [1]. The storage of waste in landfills can cause lead leaching and is a danger to the environment [2]. The requirements on discarded electrical and electronic equipment were introduced in 2003 by the European Parliament with the directive WEEE–Waste of Electrical and Electronic Equipment (the directive was actualized later) [3]. The directive includes requirements for electrical and electronic equipment and describes its treatment. One of the main objectives of this document was to reduce the number of wastes for disposal by recycling and reuse [3].

Many different methods for CRT glass recycling have been elaborated over the years. They can be classified into two main groups: closed-loop recycling and open-loop recycling [1]. In the closed-loop group, discarded computer screens and television sets are used for the production of new CRT glass. It is a cheaper, more ecological, and more effective method of obtaining new CRT glass [1]. However, nowadays, in the age of liquid crystal displays (LCD), light-emitting diodes (LED), and plasma display panels (PDP), the production of CRT glass is limited and, therefore, closed-loop recycling is no longer adequate. This is the reason why significant attention is being paid to open-loop recycling, in which CRT glass wastes are used in other branches, which gives a wide range of possibilities. CRT glass can be used for fabrication of the low-density foam glass used for thermal and acoustic insulation [4], for glass ceramics [5], in ceramic glaze formulations [6], and as an addition to clay bricks and roof tiles [7].

The serious problem concerning recycling of CRT glass is the high lead content. A typical cathode ray tube monitor consists of three parts: the neck, funnel, and screen. The content of a lead in the neck and funnel glass is in the range of 22–28%, while the screen glass is free of lead [8]. Therefore, one should consider the lead aspect, especially when using the neck or funnel glass or the mix of glasses from all three parts. Scientists developed a few methods for neutralization or complicated removal of lead from the glass matrix. According to [9], the self-propagating high-temperature synthesis (SHS) technique allows for stabilizing heavy elements (including lead) in the CRT glass. Another paper [10] describes the successful process of removing lead from discarded CRT glass with the use of subcritical hydrothermal treatment, which allows converting glass into a chemically active layered silicate compound. The majority of lead is then removed by immersion in the dilute nitric acid.

### 1.2. Application of CRT Glass in the Concrete

The exploitation of discarded CRT glass as an addition to concrete in the building industry is a separate, wide-ranging subject. The scientific literature describes both properties of the concrete mixture and finished concrete elements containing CRT glass. Utilization of CRT glass in the building industry allows both for recycling of this material and for saving natural resources. The resemblance between the chemical structure of CRT glass and sand enables their conversion in cement mortars and in concrete [11]. The majority of researches established a decrease in compressive, flexural, and tensile strength along with an increase in the amount of CRT glass in the mixture. According to the different studies, the total replacement of natural aggregate with a CRT aggregate can cause a decrease in compressive strength by 10–27% [12,13,14,15,16], a decrease in flexural strength by 26–40% [13,14], and a fall in splitting tensile strength by 10–23% [12,15,16]. CRT glass was used as a replacement of fine aggregate in most studies [12,13,14,15] and as a replacement of a coarse aggregate in one case [16]. There are also papers reporting an increase in compressive and flexural strength after overall or partial (100% or 80% by weight) replacement of natural fine aggregates (sand) with CRT glass [17]. Some works showed that the partial replacement of a conventional aggregate with CRT glass can enhance the compressive strength of concrete [18,19]. Nevertheless, regardless of declines in performance, the strength of a geopolymer containing only CRT glass as an aggregate was high enough for building applications. Depending on the composition of a mixture, the compressive strength of elements ranged from 20 MPa to 52 MPa [12,13,14,16,17]. The increase in CRT glass content causes an increase by 2–4% in the density of the concrete [12,13,14]. The addition of CRT glass increases the workability of the cement mortar [14,16,20], decreases shrinkage [12,20] and water absorption [15,20], and increases the chloride penetration resistance [21]. Many researches indicate an increase in expansion due to the alkali–silica reaction [20]. Advanced tests showed that concrete with the addition of CRT glass has X-ray radiation-shielding properties [13]. Such properties made concrete with CRT glass useful for shielding applications, for example, in CT scanner rooms or in diagnostic X-ray [13].

### 1.3. Geopolymer: An Environmental Friendly Material

Geopolymers are relatively new materials that are claimed to be an environmentally friendly alternative for concrete. Geopolymers are inorganic binders derived from the chemical reaction between alumino-silicate oxides and alkalis [22]. There are many different sources of silico-aluminates that are used as a base (a precursor) for geopolymers, such as fly ash, blast furnace slag, metakaolin, calcined clays, steel slag, and red mud [23]. The topic of eco-friendly materials is recently garnering the interest of scientists all over the world. Among the most important benefits of eco-friendly materials, scientists list the use of natural materials, a reduction in greenhouse emissions, a reduction in energy needed during manufacturing of materials and during use in construction, the possibility of the reuse of industrial wastes, and the limitation of transport by using locally available materials [24,25,26,27]. Geopolymers satisfy several out of the mentioned criteria allowing for savings in greenhouse gas emissions [28], for the reuse of different type of wastes such as mining wastes [29] and industrial and agricultural wastes [30], as well as for the utilization of hazardous wastes such as red mud [31]. Moreover, geopolymers present some superior properties such as high compressive strength, good fire resistance, acid attack resistance, and high durability [32]. Of course, geopolymers are only one proposition from a variety of eco-friendly materials that are examined and used nowadays. Ayzenshtadt et al. [33] presented the advantages of nanostructured wood mineral composites, which is created by filling the free gaps within a wood fiber structure with a special filler (i.e., nanosized basalt). Such a treatment provides better strength and sound insulation; lower water absorption; improved resistance to heat, fire, and abrasion; and negative biological impact. The addition of bio-based fibers is a topic gaining popularity in recent years as well. Hemp-fibered concretes combine the properties of being light-weight, fire-resistant, and user-friendly with quite good performance. Additionally, local materials such as raw clay may be used in the process [24]. The next eco-friendly proposition for an insulating material of low thermal conductivity is a composite of gypsum reinforced with cork fiber and cardboard waste [26]. Feduik [25] presented a way to reduce the water absorption and water-vapor permeability of fiber concrete by incorporating a fly ash, cement, and limestone composite binder subjected to a special grinding process. Some works combined geopolymers with the other environmental friendly materials such as in the case of foamed metakaolin-based geopolymers with wheat straw fibers for insulating properties [27]. Generally, the amount of different ecological solutions is promising and encourages further investigations in that field of science.

### 1.4. Application of CRT Glass in the Geopolymer

The number of studies on the utilization of CRT glass during geopolymer production is limited, especially when it involves the reuse of CRT glass in the form of an aggregate. Recent works concern the possibility of CRT glass powder addition to the geopolymer mixture preferably. Long et al. [34] researched the possibility of utilization of CRT glass as a partial replacement of the precursor in alkali-activated slag mortars. According to the tests, the presence of CRT does not affect the compressive strength severely. The specialistic analysis did not show any new geopolymerization products or a change in the structure of the products. Due to the leaching test, the addition of CRT glass below 50% allows for fulfilling the regulatory limit concerning lead leaching. The successful attempt of immobilization of powdered CRT glass with the lead content inside a fly ash-based geopolymer was reported by [35]. According to [35], the early compressive strength decreases with an increase in CRT content, but the long-term compressive strength is slightly improved. The tests conducted on a metakaolin clay-based geopolymer showed that the addition of CRT glass increases early- and long-term compressive strength, workability, and density but decreases water absorption [36]. For maximal CRT glass content equal to 20%, the requirements concerning leachable Pb were fulfilled [36]. According to the most recent studies reported by Carrillo et al. [37], even up to 20% (with respect to the weight of metakaolin) of CRT glass of sizes 0.01 mm to 1.10 mm can be incorporated into the geopolymer matrix in a way that is (according to the leaching test) safe for the environment. Scientists indicate that low alkaline conditions promote immobilization of toxic metals.

Microscopy analysis indicates that the glass surface reacts with the alkaline activator over time, which results in better binding between glass and the geopolymer matrix than between the geopolymer and sand [38,39]. Some studies report that the smooth surface of glass can cause inadequate bonding between glass and the geopolymer matrix and can lead to reduced strength especially in the short-term. The same studies indicate an increase in long-term strength, which is explained by the dissolution of fine glass particles in alkaline condition over time. The dissolved glass particles provide the additional silica with improvements in the gel network structure [40]. On the other hand, the strength can be affected by the increased number of internal voids caused by angularity of the glass particles [40]. According to [41], the strength of the geopolymer-incorporating glass aggregate depends on the grading of glass particles. Better grading leads to a reduction in pore amount and to higher strength.

The main goal of this work is to describe the behavior of a metakaolin-based geopolymer with the addition of discarded CRT glass as an aggregate. This solution would allow for the production of an environmentally friendly material, a geopolymer, and simultaneously would allow for utilization of discarded CRT glass without the necessity of powdering. In the future, the measurement of humidity inside the described geopolymer is also planned. Information about the temperature and moisture inside the material (and later inside the structure) is helpful during description of the hardening process. In the future, this information about temperature and moisture can be used for monitoring deterioration of the mechanisms inside the structure [42].

## 2. Materials and Methods

### 2.1. Materials

All mixtures were composed of metakaolin, crushed discarded CRT glass, sodium silicate, and sodium hydroxide.

**Figure 1:**
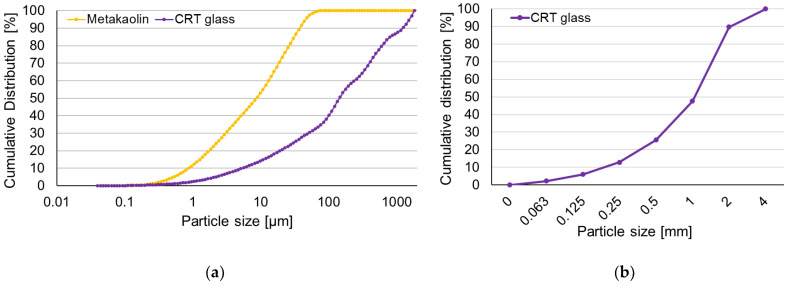
(**a**) The particle size distribution of the fine grains of CRT glass and metakaolin and (**b**) the particle size distribution of CRT glass.

Metakaolin (Astra MK 40) was used as a raw material. The material was not subjected to any special pretreatments before the tests. It was used in the same form in which it was obtained from the producer. The chemical composition is shown in Table 1. The particle size distribution of the metakaolin (performed with the use of a laser particle analyzer) is presented in Figure 1.

CRT glass played the role of a fine aggregate. CRT glass was delivered by a company collecting and recycling old CRT TV screens and computer monitors and consisted of all types of CRT glass mixed together. Firstly, the glass was separated from the rest of the kinescope and then crushed. The glass was delivered already in a crushed form (particle size > 4 mm). It was not subjected to any additional treatment before the tests. Curves presenting the size distribution of the CRT glass particles are given in Figure 1. The chemical composition (shown in Table 1) was determined by X-ray fluorescence (XRF) analysis according to the standard PN EN 15309:2010 [43].

A mixture of water solutions of sodium silicate and sodium hydroxide was used as an activator. The sodium hydroxide solution was prepared a minimum of 24 h before the tests by dissolving the NaOH pellets in demineralized water to obtain a solution of the desired concentration, 10 mol/L. The sodium silicate solution, according to the producer, had a ratio of SiO_2_ to Na_2_O between 2.4 and 2.6. The minimum content of oxides (SiO_2_ and Na_2_O) in the sodium hydroxide solution was equal to 39%. The sodium-silicate-to-sodium-hydroxide ratio in each mixture was the same as 2:1.

### 2.2. Determination of the Optimal Mixture Composition

To establish the optimal CRT-glass-to-metakaolin ratio, eight mixtures of various composition were prepared. Each series consisted of three samples. The same procedure was already presented in a recent paper [44]. The preliminary research on the optimal composition is presented again to provide logical continuity for the readers. Each following mixture contained different CRT-glass-to-metakaolin-mass ratios. Additionally, one mixture containing metakaolin only was prepared. The sodium-silicate-to-sodium-hydroxide ratio in each mixture was equal to 2:1. The total amount of activators was chosen in order to maintain similar metakaolin-to-activator-mass ratios and to ensure useful workability of the mixtures at the same time. The exact composition of all mixtures is presented in Table 2. Each mixture is assigned a code in which M/G stands for the metakaolin-to-glass-mass ratio. Mixture M/G 33/67, for instance, contains 33% metakaolin and 67% CRT glass by mass.

The procedure for the preparation of mixtures was the same for each of the following investigations. In the beginning, the precursor and aggregate were mixed. Separately, both activators (sodium silicate and sodium hydroxide solutions) were mixed for 5 min with the use of a magnetic stirrer. Then, all ingredients were merged and mixed with a mechanical mixer. The mixtures were placed in prismatic molds of dimensions of the edges of 40 × 40 × 160 mm. The molds were covered and placed in the climatic chamber at temperatures of 60 °C and 40% humidity. All samples were cured in the climatic chamber for the first 24 h. After this time, the samples were unmolded and allowed to rest for the curing time in the laboratory in an ambient temperature of about 20 °C. Flexural and compressive strength tests were performed after 7 days. Just before the tests, each sample was weighed and measured. The density of the geopolymer was calculated by dividing mass by the volume of each sample. Strength tests were carried out on the machine Controls^®^ Model 65-L27C12 Serial no 12,020,060 (Controls, Milan, Italy) according to the procedure described in the European standard EN 196-1 [45]. Firstly, each specimen was subjected to the three-point flexural strength test. The span length between supports was equal to 100 mm, and the force was greatest in the middle of the span. The broken halves of samples were subjected to the compressive strength test in the second step of the procedure. The compressive force was applied through metal plates of the size of the edges 40 × 40 mm. In each series, three samples were subjected to the flexural strength test and then six (two halves of each prism) were subjected to the compressive strength test. Two mixtures were chosen for the next steps of investigation on the basis of the strength tests results.

### 2.3. Influence of Curing Temperature on the Mechanical Behavior

The tests were continued on two chosen mixtures: M/G 25/75 and M/G 50/50. The samples were prepared according to the same procedure as described in Section 2.2 but subjected to different curing regimes. Each series consisted of three samples. Two batches of samples were cured for the first 24 h in the climatic chamber at elevated temperature (respectively, 40 °C or 60 °C) and humidity 40%. After 24 h, the samples were demolded and cured at room temperature in the laboratory for the rest of the curing period. The third batch was cured for the entire duration at room temperature and demolded just before testing. The tests were performed after 7 days.

### 2.4. Measurement of the Temperature and Mass Changes

**Figure 2:**
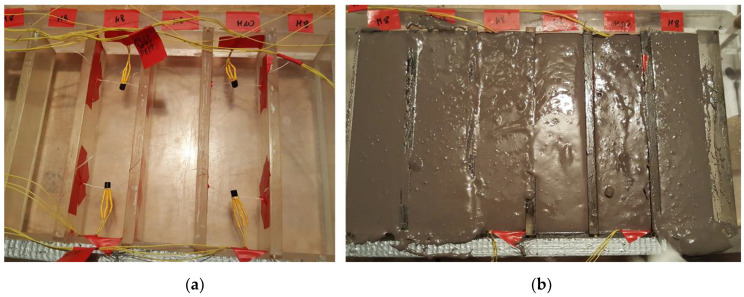
The measurement of temperature changes in samples during the curing process: (**a**) thermometers inside the empty molds; (**b**) fresh geopolymer mixture inside the molds.

The test was conducted on two mixtures: M/G 25/75 and M/G 50/50, cured at 40 °C for the first 24 h. The temperature inside the samples was measured with the use of DS18B20 thermometers by Dallas Semiconductors (Dallas, TX, USA). The thermometers were placed and stabilized on the two ends of a sample in the middle of its height and width (see Figure 2a). All steel parts of the thermometers were protected against the fresh mixture. The thermometers were connected with the electronic data-processing apparatus. The temperature was registered continuously since the moment of placing the mixture inside the molds (see Figure 2b). Two sensors were placed at the two ends in two samples made of mixtures M/G 25/75 and M/G 50/50. In conclusion, eight thermometers registered the temperature changes inside the samples and one thermometer measured the temperature in the climatic chamber.

To register the mass changes, each sample was weighed each day from the moment of demolding until the day of the test.

### 2.5. Development of Mechanical Behavior with Time

The test was carried out on mixtures M/G 25/75 and M/G 50/50, cured at 40 °C for the first 24 h. To describe the strength–time relation, flexural and compressive strength tests were conducted after 1, 3, 7, 14, and 28 days according to the standard EN 196-1 [45]. Each series consisted of three samples.

### 2.6. Atomic Absorption Apectrometry

The tests were carried out on crushed CRT glass (as a separate material) and on a sample of the M/G 50/50 geopolymer matured at room temperature. At the time of starting the assay, the geopolymer sample age was 5 weeks. Due to a comparison of the obtained results with the limit values specified in the environmental protection regulations, a broken geopolymer fitting the original dimensions of 40 × 40 × 160 mm was used. The use of a broken geopolymer fitting aimed at obtaining results adequate for the situation that could take place during practical use of the tested material (damage to the element) and that could cause, e.g., increased emission of metals to the environment.

The procedures for CRT glass and the geopolymer were the same although the materials were tested separately. The investigated material was placed in a glass vessel containing demineralized water (the mass ratio of material to water was equal to 1:10). After 1 h, the vessels were covered tightly, placed in a shaking machine, and shook for 4 h. Then, the vessels were left for 16 h in static conditions in the absence of light. After 16 h of rest, the vessels were shaken in the shaking machine for the next 4 h. After this time, sedimentation was allowed to proceed for 2 h. Then, the extract was filtered through filter paper. The extract obtained after the whole procedure was subjected to atomic absorption spectrometry.

The samples were prepared for atomic absorption spectrometry by a wet microwave digestion method. The metals in the samples were identified with the use of different types of atomic absorption spectrometry: flame, electrothermal, hydride, and cold vapor.

All analytical work connected to the chemical compound identification were carried out in a well-prepared laboratory. The air-conditioned laboratory room ensured stable and repeatable measurements. The possible impurity of samples was prevented thanks to the three-stage filtration of the supply air. Work was done with the use of two atomic absorption spectrometers made by Varian company.

Atomic absorption spectrometry is an innovative method that requires a high-quality environment to prevent errors of measurement and to ensure repeatable and reliable analysis. All requirements were fulfilled. The analysis does not exhibit significant errors.

Different methods of atomic absorption spectrometry were used for the analysis of metal content. Flame atomic absorption spectrometry (spectrometer SpectrAA-880) was used for the identification of Mn, Zn, and Cu. Electrothermal atomic absorption spectrometry (spectrometer SpectrAA-880 Zeeman) was used for identification of Cd, Pb, and V. Then, the VGA-77 vapor generation accessory was used for identification of As, Sb, and Hg, creating hydrides and cold vapors. The VGA-77 allowed for the release of volatile compounds and for the measurement of their concentrations in proper combination of an atomizer.

In addition, the following determinations were made: pH, total hardness, chlorides, total and mineral acidity, as well as general and mineral alkalinity.

The titration method was used for designation of total hardness, total acidity, mineral acidity, and total and mineral alkalinity. Chlorides were designated using the Mohr method. Calomel and glass electrodes were used for determination of pH.

### 2.7. Determination of Phytotoxicity

Phytotoxicity testing is one method of assessing the environmental impact of test substances (e.g., soil, leachate, waste, etc.). Additionally, by comparing the obtained results with those obtained for the reference substances (control test), an approximate assessment of the scale of this impact was made. Phytotoxicity tests are carried out with the use of various plants and different periods of observation of their development [46,47].

As part of the presented research, 48 h phytotoxicity tests were performed with the use of cress seeds (*Lepidium sativum* L.). Each time, 75 seeds of watercress were tested. Germinated seeds, the root of which did not reach a length of more than 1 mm, were placed on a substrate of filter paper soaked, in the first case, with distilled water (control test); in the second case, with water extract from ground CRT glass; and in the third case, with the geopolymer water extract containing CRT glass. The samples were then placed in the shade for 48 h at a temperature of 22 °C. After this time, the development of plant roots and stems was analyzed.

The M/G 50/50 geopolymer sample maturing for the entire duration at room temperature was used in the phytotoxicity determination. At the time of determination, the age of the geopolymer sample was 8 months.

## 3. Results

### 3.1. Determination of the Optimal Mixture Composition

**Figure 3:**
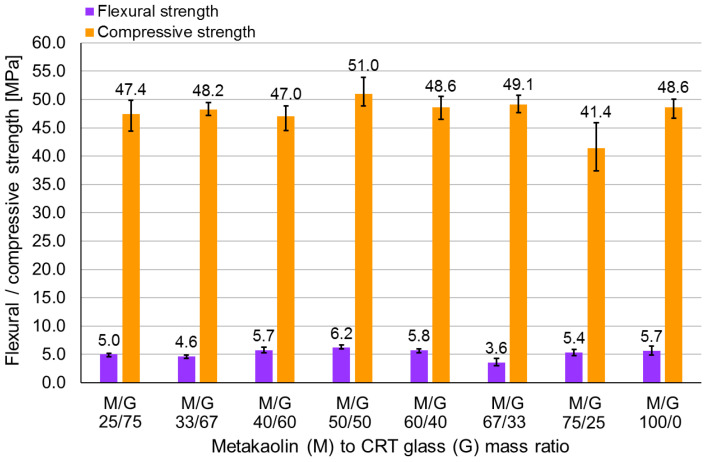
Compressive and flexural strength of geopolymer samples with varying metakaolin-to-CRT-glass ratios.

Figure 3 presents the flexural and compressive strengths of the metakaolin-based geopolymer with different CRT glass contents. The bars as well as the numbers above them represent the average from all results of each series of samples. The black segment represents the lowest and the highest results of strength within each series. According to Figure 3, there is no monotonic dependence between CRT glass content, and flexural and compressive strengths. A geopolymer made of mixture M/G 75/25 obtained significantly smaller compressive strength than the rest of samples, while the series labelled M/G 67/33 achieved the smallest flexural strength. Samples from the series M/G 50/50 showed the highest flexural and compressive strengths. Since the CRT glass content was not the only factor varying the following series (the ratio of activators use in the mixture was also varying), the obtained results were considered as results of the eight different mixtures. However, nonetheless, the varying CRT content was the most representative and significant feature of the preliminary mixtures. Two mixtures were chosen for further tests: M/G 25/75 and M/G 50/50. The first mixture was chosen due to economic reasons since it contains the most CRT glass, which allows for the most efficient recycling of waste. Mixture M/G 50/50 was chosen since it provides samples of the highest strength.

**Figure 4:**
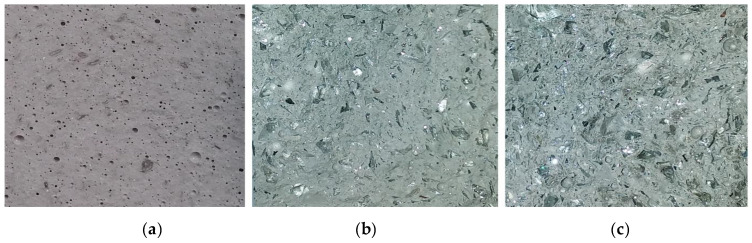
The cross section of a broken sample: (**a**) containing only metakaolin, (**b**) containing 50% CRT glass, and (**c**) containing 75% CRT glass.

According to Table 3, the density increases along with the increase in CRT glass content. The most visible difference in density (considered only between the following series) was observed between samples containing 25% CRT glass (M/G 75/25) and samples without the CRT glass content (M/G 100/0). The loss in density was equal to ≈11%. A considerable difference was registered as well between series M/G 25/75 and M/G 33/67 (≈6%) and between M/G 50/50 and M/G 60/40 (≈8%). The density differences between the rest of series ranged from 1% to 3%. Each density from the table is the mean value obtained from three determinations (all samples from each series). Figure 4 shows the cross section of broken samples of each material tested.

### 3.2. Influence of Curing Temperature on the Mechanical Behavior

**Figure 5:**
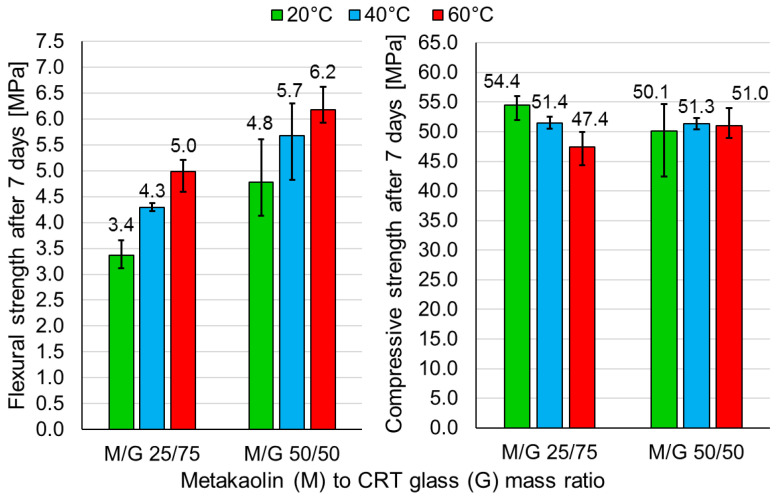
Flexural and compressive strengths of geopolymers of different metakaolin-to-CRT-glass ratios cured at different temperatures.

The influence of the curing temperature on the flexural and compressive strengths of the samples made of mixtures M/G 25/75 and M/G 50/50 is shown in Figure 5. According to the graph, the flexural strength of samples made of both mixtures increases along with the increase in curing temperature. In the case of compressive strength, the dependence between strength and curing regime is not as clear as in the case of flexural strength. The monotonic decrease in strength (respectively, by ≈6% and ≈8%) along with the increase in curing temperature was observed in the case of samples from the series M/G 25/75. In contrast, samples made of mixture M/G 50/50 achieved almost the same results in the curing temperature (the difference between the highest and the smallest compressive strength was ≈2.5%). Further tests were conducted on samples cured for the first 24 h at 40 °C.

According to Table 4, the curing temperature has an influence on geopolymer density. In the case of series M/G 25/75, the density decreases monotonically with the increase in curing temperature; however, the difference between samples cured at room temperature and at 40 °C (≈6%) is considerably higher than that between samples cured at 40 °C and 60 °C (≈1%). In the case of a geopolymer made of mixture M/G 50/50, the visible decrease in density (≈7%) was observed between samples cured at room temperature and at 40 °C, whereas samples cured at 40 °C had slightly lower density than those cured at 60 °C. The big difference between the density of samples cured at 20 °C and the density of samples cured at elevated temperatures can be explained by the fact that the first one was kept in molds until the day of testing so water evaporation was prevented. The mean values obtained from three determinations (all samples from each series) are presented in Table 4.

### 3.3. Measurement of the Temperature and Mass Changes

**Figure 6:**
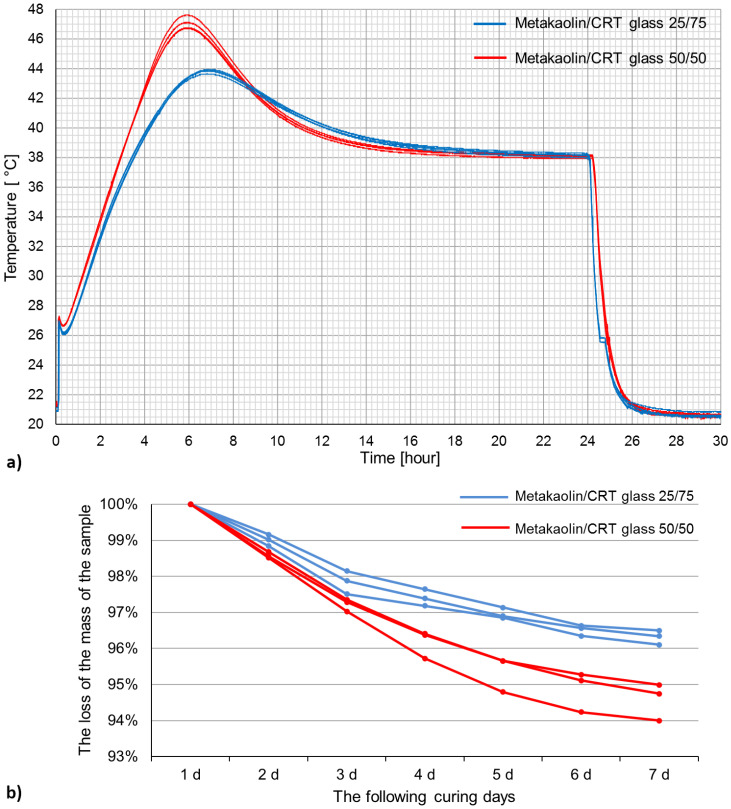
(**a**) The temperature changes in geopolymer samples during the first 30 h of curing; (**b**) the loss in mass of the samples containing 50% and 75% CRT glass during the following curing days.

The temperature change graph is presented in Figure 6. Figure 6 presents the first 30 h of curing, until the moment when the temperature inside the samples is the same as the air temperature in the laboratory.

The temperature change can be divided into several stages. The temperature registered by thermometers starts to growth immediately after placing the mixture in the molds. It achieves the local maximum and starts to decrease before the molds are placed in the climatic chamber. At the moment when the forms are placed inside the chamber, the temperature inside the samples stabilizes and becomes constant. Then, the temperature starts to increase until it reaches its maximum value. In the next step, the temperature decreases again to the temperature inside the climatic chamber. After 24 h, at the moment when the samples are taken out of the climatic chamber, the temperature instantaneously drops until it reaches room temperature (the temperature in the laboratory).

In general, the temperature gained higher values inside the geopolymer samples containing metakaolin and CRT glass in equal mass ratio. Except for the first growth and the last decrease, which take place simultaneously, the temperature rises and falls more quickly in samples made of mixture M/G 50/50. Changes in samples containing more aggregate (75%) occur gradually.

At the moment of placing the mixture in the molds, the temperature increases rapidly. After 1.5 min, it reached 26.81 °C in mixture M/G 25/75 and 27.23 °C in mixture M/G 50/50. Then, after 12 and 10.5 min, respectively, in mixtures M/G 25/75 and M/G 50/50 from placing the mixture into the molds, the temperature reduced to 26.14 °C in mixture M/G 25/75 and to 26.67 °C in mixture M/G 50/50. The temperature remained stable for 6.5 min and, after that period, started to increase steadily. In the case of samples made of mixture M/G 25/75, the maximum temperature (43.80 °C) was reached after 386 min of curing (about 6.5 h). Samples made of mixture M/G 50/50 reached the maximum temperature (47.06 °C) after 342 min of curing (almost 6 h). After reaching the top value, the temperature decreased steadily and stagnated at 38 °C (in about the 19th hour of curing). Twenty-four hours since placement into the molds, the samples were taken out of the climatic chamber and unmolded. That moment is visible in the graph in the form of a significant drop. After about 1.5 h, the temperature started to stabilize, but it was still falling until it reached room temperature (about 20.5 °C).

Figure 6 presents the mass loss of all geopolymer samples cured for 7 days. Within 7 days, samples containing more aggregates lost less mass (about 3.7%) than samples containing equal amounts of aggregates and metakaolin (about 5.4%). The mass loss is caused mainly by water evaporation. The reason for the differences in mass loss is probably caused by the fact that sample M/G 25/75 contained 18.3% activators while sample MG 50/50 contained 27.3% activators. In consequence, sample MG 50/50 contained more water at the beginning of the curing process. In the case of sample MG 50/50, the mass loss is almost monotonical by the whole curing process. Sample M/G 25/75 lost most of its mass during the first 3 days. Later, the process slowed down.

Before the strength tests, all samples were weighed. The mass of each sample was used its volume to determine the density. The geopolymer containing 75% CRT glass has a considerably greater density (2170 kg/m^3^ after 1 day and 2080 kg/m^3^ after 7 days) than the geopolymer containing 50% of this aggregate (2030 kg/m^3^ after 1 day and 1920 kg/m^3^ after 7 days. Such a result was expected since the CRT glass has a much bigger density that metakaolin.

### 3.4. Development of Mechanical Behavior with Time

**Figure 7:**
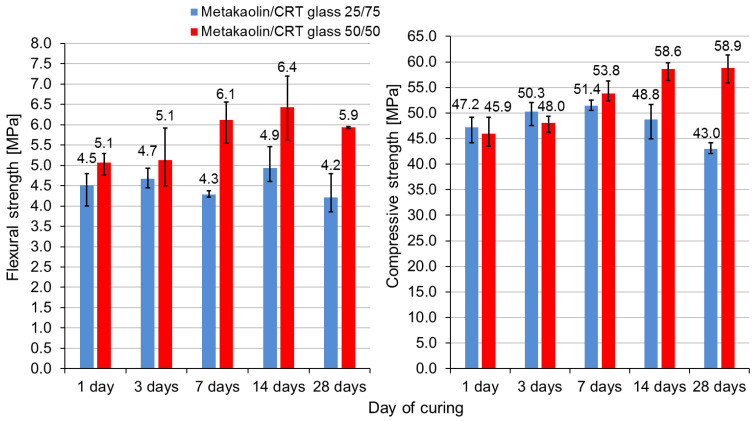
Flexural and compressive strengths of geopolymer cured for 1, 3, 7, 14, and 28 days.

Figure 7 presents the changes in the flexural and compressive strength–time relation. Flexural and compressive strength tests were performed after 1, 3, 7, 14, and 28 days. The blue bars represent samples containing 25% metakaolin and 75% CRT glass, while the red bars represent samples containing 50% metakaolin and 50% CRT glass. In the case of the geopolymer containing 50% CRT glass, the flexural and compressive strengths increased with time except for samples tested after 28 days, which obtained slightly smaller flexural strengths. The behavior of the geopolymer containing 75% CRT glass is not monotonic with time. Flexural strength after 7 days was smaller than after 1 day and 3 days. The compressive strength grew throughout the first 7 days but then decreased slightly on the 14th day. The maximum flexural (6.4 MPa) strength was obtained by samples made of mixture M/G 50/50 after 14 days since sample preparation. The maximum compressive strength (58.8 MPa) was obtained by samples made of mixture M/G 50/50 after 28 days since sample preparation. Sample M/G 50/50 in each curing day obtained higher flexural strength than samples made of mixture M/G 25/75. In contrast, samples from series M/G 25/75 had higher compressive strength after 1 day and 3 days of curing than samples from series M/G 50/50. Later (after 7, 14, and 28 days), the dependence reversed and samples made of mixture M/G 50/50 obtained higher compressive strength. The compressive strength of geopolymer samples containing 75% CRT glass shows a tendency to decrease with time.

Table 5 presents the densities of geopolymers containing 50% and 75% CRT glass measured after 1, 3, 7, 14, and 28 days. Generally, independently from the age of samples, those containing more aggregates were heavier. For all samples, the density decreased with time. In the case of both series, the most significant drop in density was observed between the 1st and 3rd days of curing, which is convergent with the results described in Section 3.3.

### 3.5. Atomic Absorption Apectrometry

Table 6 contains the results of the physicochemical analysis of aqueous extracts coming from crushed CRT glass in powdered form (considered as a separate material) and metakaolin-based geopolymers containing CRT glass, M/G 50/50. The test results were compared with Polish regulations (harmonized with EU regulations) that define the maximum permissible values of individual parameters of wastewater discharged into water or soil [48]. Not all parameters, the determinations of which were performed in the presented research, are included in the regulation [48]. However, the limit values of these parameters may be regulated by the regulations of other countries and the presented values of these parameters may be useful in assessing the impact of CRT glass and polymers on the environment. Therefore, these values are also included in Table 6. In the cases of Ni, Cr, Co, Cd, and Mn, the concentration values in both tested extracts were lower than the lowest possible to be determined by the measuring method used. At the same time, in the case of the first four, they were lower than that provided for in the regulation [48] (in the case of Mn, the permissible concentration was not specified in the regulation). Additionally, the concentrations of Fe, Cu, and Zn in the water extracts did not exceed the permissible values. In the case of Pb, its concentration in the CRT glass water extract exceeded the limit value by more than 3 times. However, in the case of a water extract of geopolymer, the concentration values of this element are more than 4 times lower than the limit values.

Comparing the concentrations of metals in water extracts (with concentration values higher than the threshold of the method applicability), it was found that the concentrations in the geopolymer water extract are lower: from two (Cu) to almost fourteen times (Pb). Low leachability of Pb from geopolymers was also confirmed in other studies (e.g., [24]). The above results indicate a partial immobilization of metals in CRT glass in geopolymers and the reduction of their transmission to the environment as a result (similar conclusions are presented, e.g., in [27]). Unfortunately, the pH values of both tested water extracts are outside the range specified in the regulations. The reaction of the CRT glass water extract is close to the lower limit of the acceptable range.

In the case of most of the parameters analyzed above, their values found in the case of the geopolymer water extract are lower than in the case of the CRT glass water extract. However, a different situation is observed in the case of pH, and general and mineral alkalinity.

### 3.6. Phytotoxicity Test

Table 7 shows the results of the phytotoxicity determinations. The table shows the mean values and standard deviations of the growth of the cress root and stem (mm), both for the control sample and for the tests using water extracts of CRT glass and geopolymer containing this glass. Moreover, the table shows the results of the sprouting index calculations for the trials with the use of water extracts (calculated for the root and stem).

The germination index was determined according to the following formula:

(1) $$I_{g}=\frac{n\cdot L_{z}}{n_{c}\cdot L_{c,z}}$$

where *n* is the number of seeds in the test sample, *n_c_* is the number of seeds in the control sample (in the presented tests, *n* = *n_c_*), *L_c,z_* is the mean length of the cress root/stem in the control sample, *L_z_* is the mean length of the root/stem in the test sample, and *z* is the root/stem.

It should be noted that, in both the control and both aqueous extracts, an increase in all cress seeds was observed.

As a result of the phytotoxicity tests, it can be concluded that the CRT glass water extract, compared to the control sample, has stimulating properties (both stem and root development). On the other hand, the water extract of the geopolymer containing CRT glass shows inhibitory properties (both the development of the stem and the root). This is probably the result of the reaction of this extract.

## 4. Discussion

### 4.1. Determination of the Optimal Mixture Composition

There is no consensus among scientists regarding the influence of CRT glass content on the strength of geopolymers. Moncea et al. [49] derived that the incorporation of ≈21% of CRT glass (by mass, where fly ash and CRT glass are at 100%) to fly ash-based geopolymers caused an increase in compressive strength by 6% in comparison to the sample without CRT glass. The addition of ≈42% of CRT glass caused, in turn, a decrease in compressive strength by 22% in comparison to the control sample. In contrast, in the case of a slag-based geopolymer, the incorporation of ≈21% and ≈42% of CRT glass caused a decrease in compressive strength by 2% and 6%, respectively, in comparison to the control sample based on slag only. According to Badanoiu et al. [50], the highest compressive strength was obtained by samples containing powdered CRT glass only. There was no significant difference between samples containing 25%, 50%, and 75% of fly ash. The compressive strength of samples containing fly ash was more than two times lower than that of samples with pure CRT glass. In contrast, Long et al. [34] reported that both flexural and compressive strengths of slag-based geopolymers decreased monotonically with the increase in CRT glass. However, the drop is not linear. In the case of the first four mixtures containing 0%, 10%, 30%, and 50% of CRT glass by mass, both flexural and compressive strengths decreased linearly by about 5–7%, whereas in the case of the mixture containing 70% CRT glass, the rapid drop in both flexural and compressive strengths by about 18% was noticed. Scientists explain that the pozzolanic activity of CRT glass is lower than the pozzolanic activity of slag. As an effect, the geopolymerization process is slower and less geopolymerization products are present in a matrix that has repercussions on the strength of a hardened material [34]. Tomas Opletal [51] also reported a decrease in compressive strength with an increase in CRT glass content. Regarding two different series, compressive strength dropped by 11% and 6% when the CRT-glass-to-metakaolin ratio increased from 3:1 to 7:1 and from 2:1 to 3:1, respectively.

No clear dependence between CRT glass content and strength was registered. It should be emphasized that CRT glass content was not the only variable. The activator content was altered as well to provide sufficient workability. The changing activator-to-metakaolin ratio could alter the dependence between CRT glass content and strength. Nonetheless, on the base of the obtained results, it was decided that, among the tested mixtures, those containing 75% (M/G 25/75) and 50% (M/G 50/50) CRT glass are the best ones. Samples made of both mixtures characterized good flexural and compressive strengths. A mixture containing 75% CRT glass is more environmentally friendly since it allows for better utilization of waste. It demands less activators as well since it contains less metakaolin. Mixture M/G 50/50, in turn, provided samples with the best flexural and compressive strengths among all tested series. Furthermore, this mixture also allows for utilization of significant amounts of CRT glass.

### 4.2. Influence of Curing Temperature on the Mechanical Behavior

The observed increasing tendency of flexural strength along with the increase in curing temperature is convergent with the observations presented by Alonso et al. [52]. However, Alonso et al. indicated an abrupt and high (almost 100%) growth in flexural strength with the increase in curing temperature from 35 °C and 45 °C, while the further gain in strength for samples cured at 65 °C is only about 15%. In this paper, the increase in flexural strength is rather linear. Sun et al. [53] also reported an increase in flexural strength of metakaolin-based geopolymers along with the increase in curing temperature within the first days of curing. In contrast to Alonso et al., a much more significant difference was observed between flexural strength of samples cured at 40 °C and 60 °C than between the flexural strength of samples cured at 20 °C, 30° C, and 40 °C. Sun et al. indicated that, on the 7th day of curing, the compressive strength monotonically increased with the increase in curing temperature. The overall increase between samples cured at 20 °C and samples cured for the first 24 h at 60 °C is equal ≈9%. The monotonic increase in compressive strength for the increase in curing temperature in the range 20 °C to 60 °C was also observed by Mo et al. [54]. Scientists report an overall increase in compressive strength by ≈58% after 7 days within that range of temperatures. However, for higher curing temperatures (80 °C and 100 °C), compressive strength starts to decrease. Mo et al. derived that elevated curing temperatures promote dissolution of precursors from amorphous phases and, as an effect, accelerates the formation of rigid and hard matrices. An elevated temperature is crucial at the early stages of geopolymerization. High temperatures provide sufficient amounts of gel, which bonds tough metakaolin particles. However, too high a curing temperature may lead to the situation where polymerization takes place too fast and some metakaolin particles remain undissolved and captured by the geopolymer gel, which prevents further dissolution and, finally, forms hard and compact structures [54]. The results presented by Sun et al. and Mo et al. are divergent with the results presented in this paper, where compressive strength is approximately equal to or decreases along with the increase in curing temperature, dependent on the mixture composition. More similar results are shown by Ekaputri [55], who did not observe any significant differences between the compressive strengths of samples cured at 25 °C, 40 °C, 60 °C, and 80 °C. Rovnanik [56], in turn, reported approximately equal 7th day compressive strengths of samples cured at 20 °C and 40 °C and significantly (≈17%) lower strengths of samples cured at 60 °C and 80 °C. A similar dependence was noticed in the case of flexural strength. According to Rovnanik, when geopolymer was cured at lower temperatures, the geopolymerization process was slower, which allowed for the development of less porous, more tough, and stronger structures of better quality. As in the case of this paper, Rovnanik noticed that the density of the geopolymer has a tendency to decrease along with the increase in curing temperature.

According to the results presented in this paper, the elevated curing temperature enhances the flexural strength and has no crucial impact on compressive strength. It was decided to continue the tests on samples cured for the first 24 h at 40 °C. In the case of both mixtures, samples cured at 40 °C achieved high flexural and compressive strengths. Additionally, curing at 40 °C is more environmentally friendly and economical than curing at 60 °C. In contrast, curing at room temperature demands keeping the samples in molds for longer times (here, 7 days); otherwise, the samples can be affected by cracking (it was observed by previous tests [44]). However, two remaining curing procedures have advantages as well. Curing at 60 °C provided the highest strength results in the case of series M/G 50/50 and the highest flexural strength results in the case of series M/G 25/75. Curing at 20 °C, in turn, is the most environmentally friendly. The economic issue is complex because a low curing temperature is counterbalanced by the necessity for longer curing in the molds. The planned future tests will cover, among others, a comparison of long-term strength development of samples cured at different conditions.

### 4.3. Measurement of the Temperature and Mass Changes

During the measurement of temperature changes in a metakaolin-based geopolymer, Davidovits et al. [57] obtained a similar shape of the temperature curves; however, the first peak was not registered. The maximum temperature of samples cured in 40 °C was reached after 4 h and was equal to about 70 °C, which is earlier and more than in the case of the results presented in this paper. According to Yao et al. [58], the first exothermic peak begins at the moment of mixing metakaolin with the activators and is caused by instant absorption of the activators on the surface of the metakaolin particles. Then, the reactions slow down but continue. Further heat release is connected with complete dissolution of the metakaolin particles and with the polymerization process [58].

The results presented in this paper indicate that the increase in temperature inside the tested material is rather low. The results are promising due to the possible shrinkage appearance. In the future, the authors will continue tests connected with the development of the temperature inside a metakaolin-based geopolymer with CRT glass during the curing process. Tests should be repeated in the case of much bigger elements. The authors are planning to determine the temperature inside samples cured at different temperatures as well and to determine with high accuracy the humidity changes.

### 4.4. Development of Mechanical Behavior with Time

In general, the other scientific researches confirm the tendency of the strength of the metakaolin-based geopolymer to increase with time. Research done by Mo et al. [54] showed that compressive strength of a metakaolin-based geopolymer, cured at temperature 40 °C, increases with time for the first 7 days. The growth rate of the compressive strength was bigger than in the paper presented above. The compressive strength between the 1st and 3rd days increased by about 23% and, between 3rd and 7th days, by about 29%. The increase in compressive strength of metakaolin-based geopolymer cured in 40 °C was also registered by Ekaputri et al. [55]. Scientists noted that compressive strength increased rapidly between the 3rd and 7th days of curing. The difference in strength between the 7th and 14th days is negligible. Compressive strength measured on the 21st and 28th days was again significantly higher. Rovnanik [56] reported that both the compressive and flexural strengths of a metakaolin-based geopolymer generally increases with time but that the growth is dependent on the curing time in elevated temperatures (40 °C). In all cases, the biggest growth in strength was noticed between the 1st and 3rd days of curing. Sun et al. (2019) [53] reported a monotonic increase in both flexural and compressive strengths of metakaolin-based geopolymer samples cured for 3, 7, 14, 28, 96, 180, and 365 days. The biggest growth in flexural strength took place between the 3rd and 7th days of curing, while the compressive strength increased most rapidly between the 7th and 14th days of curing. In the case of this paper, the biggest increase in both flexural and compressive strengths was observed between the 3rd and 7th days of curing. Sun et al. (2019) [53] reported that, generally, the biggest gain in strength took place within the first 28 days of curing, in the cases of both flexural and compressive strengths.

During the tests presented in the paper, the metakaolin-based geopolymer with CRT glass generally gained strength with time. Such a tendency is promising, especially when considering a metakaolin-based geopolymer with CRT glass as a prospective building material. However, tests verifying long-term mechanical behavior are needed, especially in the case of a geopolymer containing 75% CRT glass, which showed a tendency to lose strength (it was especially visible in the case of compressive strength) with time. Long-term studies should be carried out to find out if strength stabilizes finally at an acceptable level. If not, this mixture cannot be considered a building material. Until the nature of the drop in strength is revealed and its further tendency is determined, no extended tests are planned for a geopolymer made out of mixture M/G 25/75.

### 4.5. Atomic Absorption Apectrometry and Phytotoxicity Test

A comparatively big difference between the content of Pb in an extract from unstabilized soils (CRT powdered glass and raw material) and a geopolymer containing CRT glass is reported by Ogundiran et al. [36]. Introducing CRT glass into the geopolymer mixture has not been very popular so far, but a lot of scientists successfully encapsulated separate heavy metals within the geopolymer matrix [37,59,60,61,62,63]. Many scientific investigations prove also similar capabilities of concrete for the encapsulation of lead from CRT glass and substantial reductions in Pb leaching [16,18,19,34].

The presented results show that using the CRT glass as an aggregate in geopolymers can significantly lower leaching of toxic metals from this hazardous waste. It is also important that, in the case of the geopolymer water extract, the concentrations of the determined metals were lower than the limit values specified in the regulations [2].

The only parameter that did not fall within the regulatory range for both CRT glass and geopolymer water extracts was pH. At the same time, the obtained pH result for the water extract obtained from the geopolymer is similar to the results presented, for example, in [64], where for 28 day geopolymers (using CRT glass), the obtained pH was about 10.5 and 11.5.

It can be assumed that the relatively high pH value of the geopolymer water extract was the main reason for the inhibitory properties of this extract in the phytotoxicity determination.

Summing up, it should be noted that, despite the observed decrease in the leaching of metals from CRT glass, which is a component of geopolymers, the overall assessment of geopolymer impact on the environment still needs to be continued and extended.

## 5. Conclusions

The paper presents the results of an investigation on the mechanical behavior of a metakaolin-based geopolymer with waste CRT glass in the role of an aggregate. The following issues were considered: the influence of CRT glass content and curing temperature on mechanical behavior, changes in the geopolymer temperature with time, changes in geopolymer density with time, changes in flexural and compressive strengths, and physicochemical analysis of the aqueous extracts. The following conclusions were drawn:

1. No strict dependence between CRT glass content and geopolymer strength was observed. The obtained flexural strength ranged from 3.6 to 6.2 MPa (samples containing metakaolin-to-CRT-glass ratios of 2:1 and 1:1, respectively), while the compressive strength ranged from 41.4 to 51.0 MPa (metakaolin-to-CRT-glass ratios of 3:1 and 1:1, respectively).
2. Mixtures containing metakaolin-to-CRT-glass-mass ratios equal to 1:1 and 1:3 were considered optimal taking into account environmental, economic, and mechanical issues. However, the flexural and compressive strengths of the geopolymer with a precursor-to-aggregate ratio of 1:3 showed alarming tendency to decrease with time (compressive strength decreased by over 16% within the 7th and 28th days of curing). The strength of the geopolymer containing a metakaolin-to-CRT-glass-mass ratio equal to 1:1 showed promising tendency to increase with time.
3. The curing of samples for the first 24 h at 40 °C was decided as optimal due to the high strength and possibility of quick demolding. Samples containing a metakaolin-to-CRT-glass ratio of 1:3 achieved after 7 days a flexural strength of 4.3 MPa and a compressive strength of 51.4 MPa, while samples with an ingredients ratio of 1:1 obtained strengths equal to 5.7 MPa and 51.3 MPa, respectively. After just one day, the compressive strength of the geopolymer made out of both mixtures exceeded 45 MPa.
4. The highest temperature (47.06 °C) was registered in the samples containing more metakaolin (50% by mass) after about 6 h of curing. The loss in mass was also higher in samples containing a metakaolin-to-CRT glass ratio of 1:1.
5. The amount of chosen toxic elements in the aqueous extract from CRT glass is much higher than that in an extract from a geopolymer containing CRT glass. The following elements were determined: Fe, Mn, Cu, Ni, Cr, Co, Zn, Pb, and Cd. The most significant difference was observed in the case of Pb, for which the concentration in the CRT extract was almost 14 times higher than in the geopolymer. The amount of all investigated elements (including toxic ones) in the leachate from the geopolymer fulfilled the regulatory limits. Therefore, it may be concluded that the CRT glass was successfully immobilized within the geopolymer matrix.

This paper presented a new type of geopolymer as a promising material and gives a new possibility of recycling CRT glass without any special pretreatment. According to the mechanical performance, this metakaolin-based geopolymer with CRT glass in the form of an aggregate can be treated as a building material with excellent properties. In order to obtain full knowledge about the tested material, it is necessary to conduct further studies, in particular regarding long-term changes in its properties and multiparameter environmental assessment.

## Figures and Tables

**Table 1:** Chemical compositions of metakaolin ^1^ and cathode ray tube (CRT) glass ^2^.

Oxide Composition (wt %)	CRT Glass	Metakaolin
SiO_2_	76.10	53.12
Na_2_O	6.25	0.09
CaO	5.24	0.44
BaO	2.62	-
K_2_O	2.36	0.73
MgO	1.64	0.26
PbO	1.61	-
SrO	1.42	-
Al_2_O_3_	1.37	42.14
SO_3_	0.55	-
Fe_2_O_3_	0.38	0.45
ZrO_2_	0.28	-
TiO_2_	0.12	0.64
ZnO	0.05	-
As_2_O_3_	0.01	-
H_2_O^-^	-	0.22
P_2_O_5_	-	0.03
Cl	-	0.02
S	-	0.01
MnO	-	0.01

^1^ Data obtained from the producer: Astra Technologia Betonu^®^, Straszyn, Poland.^2^ Chemical composition was determined by X-ray fluorescence (XRF) analysis, performed by EkotechLAB^®^, Gdańsk, Poland.

**Table 2:** Composition of geopolimeric mixtures.

Mixture	Metakaolin (kg/m^3^)	CRT (kg/m^3^)	Sodium Silicate (kg/m^3^)	NaOH (kg/m^3^)	Si/Al ^1^	Na/Al ^1^
M/G 25/75	524	1572	314	157	1.40	0.55
M/G 33/67	657	1335	365	182	1.37	0.51
M/G 40/60	755	1133	417	208	1.37	0.50
M/G 50/50	898	898	449	225	1.34	0.46
M/G 60/40	995	663	521	260	1.36	0.48
M/G 67/33	1037	510	553	276	1.36	0.49
M/G 75/25	1078	359	586	293	1.37	0.48
M/G 100/0	1083	0	675	337	1.41	0.57

^1^ Ratios calculated for metakaolin, sodium silicate, and sodium hydroxide (without CRT glass).

**Table 3:** The average density of geopolymers with different CRT glass contents.

Samples Made of a Mixture	M/G 25/75	M/G 33/67	M/G 40/60	M/G 50/50	M/G 60/40	M/G 67/33	M/G 75/25	M/G 100/0
Density (kg/m^3^)	2090	1960	1940	1920	1770	1720	1690	1500

**Table 4:** The average density of geopolymers with different CRT glass contents and cured at different temperatures.

	20 °C	40 °C	60 °C
M/G 25/75	2240	2110	2090
M/G 50/50	2040	1890	1920

**Table 5:** Density of the geopolymers in the following curing days.

	1 Day	3 Days	7 Days	14 Days	28 Days
M/G 25/75	2200	2130	2110	2090	2070
M/G 50/50	1970	1930	1920	1900	1870

**Table 6:** Physicochemical analysis of aqueous extracts and the highest limit values.

Designation	Unit	Aqueous Extract from Geopolymer	Aqueous Extract from CRT Glass	Limit Values [48]
pH	-	11.0	6.3	6.5–9
Total hardness	mval/dm^3^	0.08	0.82	-
Chloride	gCl/dm^3^	0.0138	0.0138	1
Total acidity	mval/dm^3^	0.0	0.8	-
Mineral acidity	mval/dm^3^	0.0	0.0	-
Total alkalinity	mval/dm^3^	6.0	0.0	-
Mineral alkalinity	mval/dm^3^	10.2	0.6	-
Fe	ppm	0.12	0.42	10
Mn	ppm	<0.015	<0.015	-
Cu	ppm	0.01	0.02	0.1
Ni	ppm	<0.02	<0.02	0.1
Cr	ppm	<0.03	<0.03	0.05
Co	ppm	<0.025	<0.025	0.1
Zn	ppm	0.03	0.31	2
Pb	ppm	0.12	1.66	0.5
Cd	ppm	<0.006	<0.006	0.07

**Table 7:** Results of phytotoxicity determinations: mean values and standard deviations of the root and stem increment of cress (mm) and the value of the germination index.

	Unit	Control Sample		CRT Glass Water Extraction		Water Extract of a Polymer Containing CRT Glass	
		Stalk	Root	Stalk	Root	Stalk	Root
Average value	mm	8.9	15.5	11.9	18.2	6.6	12.9
Standard deviation	mm	3.3	4.8	3.6	5.2	2.4	11.8
Germination index	%	100	100	134.5	117.2	74.5	82.8

## Data Availability

The data are available upon request.
